# Toxic Wasting Disorders in Sheep

**DOI:** 10.3390/ani11010229

**Published:** 2021-01-18

**Authors:** Jéssica Molín, Fábio S. Mendonça, Eileen E. Henderson, Akinyi C. Nyaoke, Gustavo A. Ramírez, Mauricio A. Navarro, Francisco A. Uzal, Javier Asín

**Affiliations:** 1 Animal Science Department, University of Lleida, Lleida 25198, Spain; jessica.molin@udl.cat (J.M.); gustavo.ramirez@udl.cat (G.A.R.); 2 Laboratory of Animal Diagnosis, DMFA/UFRPE, Recife, Pernambuco 52171-900, Brazil; fabio.mendonca@ufrpe.br; 3 California Animal Health and Food Safety Laboratory (CAHFS), San Bernardino Branch, University of California, Davis, CA 95616, USA; eehenderson@ucdavis.edu (E.E.H.); canyaoke@ucdavis.edu (A.C.N.); mnavarrob@ucdavis.edu (M.A.N.); fauzal@ucdavis.edu (F.A.U.)

**Keywords:** sheep, intoxications, poisonous plants, wasting

## Abstract

**Simple Summary:**

There are several substances, either inorganic or organic that may be toxic for sheep. Intoxications by some of these substances have an acute clinical course with specific signs and lesions that may guide the diagnostic procedures. However, there are other insidious, subacute to chronic presentations, usually related to continuous intake of the toxic substance over long periods of time, that may present with emaciation, ill-thrift, and/or poor external aspect. In such scenarios, diagnosis may be challenging and should be achieved through a combination of history of exposure, subtle gross and histological findings (if present), and available ancillary tests.

**Abstract:**

Infectious and parasitic agents have been frequently associated with debilitating and wasting conditions in sheep. The prevalence of these agents has probably undermined the role of toxic causes as contributors to such disorders. In addition, many of these intoxications frequently produce acute clinical disease with specific and characteristic lesions, thus a causal relationship with the toxic substance may be relatively easy to establish. However, persistent exposure to some of these organic or inorganic toxic substances may lead to emaciation, ill-thrift, and poor external aspect. The anti-nutritional factors and alkaloids of several plants, including pyrrolizidine alkaloids, among others, have also been associated with emaciation and/or poor general performance in sheep flocks. In this review, some of these disorders are discussed with an emphasis on clinical signs and lesions, relevant diagnostic aspects, and available therapeutic approaches. In most cases, demonstrating a history of exposure should be one of the most relevant aspects of the diagnostic approach, and removing the animals from the toxic source is the cornerstone of the majority of the treatment strategies.

## 1. Introduction

There are several diseases of sheep that may cause wasting, leading to decreased productivity and economic losses in sheep flocks as a result. Johne’s disease, maedi-visna, caseous lymphadenitis, and several parasitic diseases, amongst others, have been traditionally included under the umbrella of the so-called “thin ewe syndrome”, a concept that encompasses several diseases with wasting as the main clinical sign [1,2,3,4]. Hence, this concept has included mostly infectious and parasitic disorders, probably underestimating the role of other disease causes. 

Commercial diets must be correctly formulated to prevent either toxicity or deficiencies due to excess or lack of certain mineral components, respectively [5]. There are also pernicious chemical and biological products that cause acute toxicity in the short-term, but exposure to some of them over longer periods of time may also contribute to wasting and poor general performance [6]. Certain plants, including grasses, legumes, and shrubs, contain anti-nutritional and toxic compounds that, depending on the situation, may cause deleterious effects on animals that consume them [7,8]. Some of these substances are non-lethal, but their consumption during long periods of time, especially with concomitant confinement and/or food scarcity, may lead to emaciation as well.

Here we review conditions caused by inorganic and organic toxic compounds and some poisonous plants, with an emphasis on clinico-pathologic and diagnostic aspects, and we analyze their contribution to wasting, debilitation, and/or loss of productivity in sheep.

## 2. Inorganic and Organic Toxic Compounds

This section includes several substances with toxic potential such as heavy metals and other biologically active elements. Most of them are associated with peracute or acute episodes of intoxication when animals are exposed to a single high dose [6]. In those instances, the clinical signs and lesions tend to be specific and well-known, and a shorter time frame between the exposure and the episode facilitates the diagnosis. However, those clinical presentations are not the main object of this review, and extended explanations may be found elsewhere. 

Contrarily, persistent exposure to lower doses of these compounds over time may lead to chronic intoxication, with poor general performance and variable degrees of emaciation [6]. In these cases, the diagnosis could be fastidious, since a combination of mild and/or unspecific clinical signs and lesions, history of exposure, and detection of the toxic substance are required to establish the diagnosis. The following discussion attempts to shed light on the different aspects of these insidious presentations. Available treatments for some of these intoxications are summarized in Table 1, and each compound is discussed below individually.

### 2.1. Copper

Sheep are very limited in their ability to excrete copper (Cu). Indeed, sheep tend to accumulate Cu in the liver along time, thus being extraordinarily susceptible to chronic Cu intoxication [9,10]. This can occur mainly in three forms: (i) Excessive consumption due to contamination of drinking water and/or food with Cu-containing compounds (e.g., Cu sulfate); (ii) Low levels of dietary molybdenum (Mb), which increase the rate of Cu absorption in the gut, since Mb tends to combine with sulfate and Cu, making the latter insoluble; (iii) Concomitant exposure to hepatic toxins, such as plants containing pyrrolizidine alkaloid (PA) [9]. Interestingly, chronic Cu poisoning in sheep is a long-term intoxication with an acute clinical presentation [10]. 

Cu accumulates in hepatocyte lysosomes and is actively incorporated and stored in new hepatocytes when others die and release it. However, there is a threshold level at which the organ cannot cope with this turnover rate and significant hepatocellular necrosis ensues. Subsequently, plasma Cu levels increase, causing a sudden crisis of intravascular hemolysis, anemia, and death. Sheep may show no clinical abnormalities before the hemolytic crisis occurs, although in certain cases there could be an elevation of hepatic enzymes such as plasma aspartate transaminase (AST) and gamma-glutamyl transferase (GGT), with anorexia and ill-thrift prior to this crisis [6,10]. Weight loss and hypoxaemia were detected in all sheep involved in an experimental Cu intoxication two weeks before the hemolytic crisis started [11]. 

Lesions are very characteristic once the hemolytic crisis occurs, and include widespread icterus, yellow to orange and friable liver, dark, “gunmetal-colored” kidneys, and dark red urine. Histologically, there is hepatic centrilobular necrosis and renal tubular necrosis with hemoglobin casts in the tubular lumina [9]. It is important to identify animals in which the hemolytic crisis has not started, since once this occurs the treatment has very few chances of being successful [6]. Sheep with high blood Cu may be treated with intravenous ammonium tetramolybdate to decrease the Cu:Mb ratio. Furthermore, including Mb salts to the concentrate ration may help to prevent new cases and decrease mortality in the flock [6,10,12]. Recently, it has been proposed that dietary zinc (Zn) supplementation at 300 mg/kg dry matter may prevent accumulation of Cu in the liver of sheep exposed to high concentrations of this element [11].

### 2.2. Nitrogen-Containing Compounds

#### 2.2.1. Nonprotein Nitrogen Sources

These substances encompass all non-protein sources of nitrogen (N), such as urea, from which sheep are able to synthetize proteins. Therefore, ovine rations are occasionally supplemented with urea and other similar substances to take advantage of this process [13]. Normally, the ruminal microbiota converts these N sources into ammonia, which is then used to produce amino acids and proteins. However, if the ingested amount of non-protein N exceeds the metabolic capacity of the rumen to produce proteins, these substances persist in the ruminal fluid and are turned into excessive ammonium, which increases the ruminal pH above 8. Free ammonia is also absorbed, causing hyperammonemia and toxicity. In most cases, this is an acute process related to the sudden incorporation of the N source into the diet. Excessive salivation, tremors, ataxia, and bloating are described. Subacute or chronic clinical signs are rare, and may range from mild nervous signs to lethargy, anorexia, and poor external aspect. Advanced emaciation is uncommonly observed with urea poisoning, but periods of fasting and malnutrition associated with other concomitant processes may predispose to urea intoxication [14,15].

There are no diagnostically relevant gross or histologic findings, but the demonstration of elevated ruminal pH (8 or above) and high ammonia concentration in serum and/or aqueous humor supports a diagnosis [13]. Treatment strategies are focused on reducing the ruminal pH. Vinegar, given orally or instilled directly into the rumen by gastric tubing, has been able to successfully control some clinical cases [6].

#### 2.2.2. Nitrates and Nitrites

Sheep may be exposed to nitrates by consuming nitrate accumulating plants, decaying organic matter, and/or certain fertilizers [6]. Recently, nitrate supplementation has been proposed as an effective method to reduce methane emissions by ruminants [16]. Some examples of nitrate accumulating plants and their effects are described later in this paper (see Section 3.4 Nitrate-Accumulating Plants). Nitrate-containing fertilizers may contaminate drinking water, which becomes one of the main sources of exposure for animals. Ruminal microbiota transforms nitrates into the more toxic nitrites, which are absorbed, inducing the formation of methemoglobin in blood and a subsequent decrease of tissue oxygenation. The clinical course is most commonly acute, with rapid pulse, cyanosis, and weakness. Exposure to sublethal doses of nitrates over time may lead to weight loss and reproductive problems, thus contributing to general ill-thrift and poor performance [17]. 

Necropsy findings are suggestive of nitrate intoxication if the carcass is fresh enough. A brownish discoloration in muscles, lungs, and/or brain, with brown, dense blood (i.e., “chocolate-like”) may be found. These changes are due to methemoglobinemia, and since methemoglobin rapidly turns into hemoglobin, they tend to disappear shortly after death [18]. Elevated serum, plasma, and/or ocular fluid nitrate concentrations are often diagnostic. Ocular fluid may be analyzed by qualitative nitrate strips as a screening procedure, followed by a quantitate confirmatory test in positive cases [18]. Treatment is focused on reducing methemoglobin to hemoglobin. Intravenous injection of an aqueous solution of methylene blue has proven to be effective for this purpose [6].

### 2.3. Fluoride/Fluorine

Fluoride (F) is the monovalent anion of fluorine and the form that commonly occurs in nature as part of different minerals [19]. Livestock may be chronically exposed by grazing on pastures close to industrial sites with F emissions, such as aluminum factories, or in active volcanic areas [20]. The condition is better described in cattle, but sheep might develop a similar syndrome [21]. F tends to accumulate in bones and teeth of livestock, and thus exostosis and malformations may appear in long bones and mandible, whereas teeth develop wearing and discoloration. These lesions may lead to loss of condition due to stiffness, lameness, and apprehension and chewing problems due to poor dentition. Botha et al. [22] described an outbreak of fluorosis that affected sheep and cattle. Sheep developed pitting of the enamel and abnormally elevated plasma levels of urea and alkaline phosphatase. Icelandic sheep experimentally poisoned with F for 20 weeks did not develop gross or microscopic lesions in bones or teeth, although loss of appetite and recumbency were seen [23]. Poor skin and hair coat have also been associated with chronic F exposure [19]. Elevated urine and/or plasma F concentrations together with compatible lesions support a diagnosis of fluorosis. The cornerstone of the therapeutic approach is removing the animals from the contaminated pastures. In addition, calcium or aluminum salts may be added to the concentrate to reduce F solubility and prevent its accumulation [6].

### 2.4. Lead

Persistent ingestion of lead (Pb) may occur in contaminated areas, such as those in which mining has been actively practiced over the years [24]. Ingestion of metallic objects that persistently release Pb in the prestomachs and/or old Pb-containing paints, amongst others, may also cause poisoning [6]. Chronically poisoned animals may develop progressive anorexia, weight loss, weakness, and reproductive disorders including abortions. Emaciation and other non-specific lesions are detected at necropsy. Lambs raised in areas with high environmental levels of Pb could develop osteoporosis [25]. Pb tends to accumulate in growing bones, and a band of sclerosis known as “lead line” may be detected in the metaphyses. Acutely poisoned animals tend to develop neurologic signs [6]. Histologically, acid-fast intranuclear inclusions may be observed in some osteoclasts, renal tubular epithelium, and hepatocytes [26,27]. Chronic Pb intoxication causes anemia, since the synthesis of heme group is inhibited [27]. Detecting high levels of Pb in liver, kidney, and/or blood is diagnostically relevant. Determining blood levels of Pb may also be helpful in establishing the prognosis of the intoxication in live animals [28]. No fully effective treatment exists, but after removing the animals from the contaminated areas, chelating agents such as calcium disodium ethylenediaminetetraacetic acid (CaEDTA) can be applied. Thiamine hydrochlorite has successfully reduced the deposition of Pb in tissues and is a good complementary therapy to chelation [28].

### 2.5. Selenium

Selenium (Se) is a mineral with essential functions in a variety of biological processes. Both deficiency and excess of Se can cause disease, and the range that separates deficient and toxic concentrations of this mineral is very narrow. Intoxication may occur in sheep with overdosed injections or by providing oral supplementation trying to correct a deficiency [5], although this tends to cause acute rather than chronic toxicity. Intoxication associated with oral Se supplementation is more commonly observed in lambs [29,30]. Persistent exposure to Se-contaminated water or Se-accumulating plants in seleniferous soils is more likely to cause chronic intoxication. Alkali disease refers to chronic Se toxicosis related to consumption of Se-containing pastures over time. 

Poisoned sheep may show depression, anemia, and emaciation [31]. Hoof malformations occur in cattle and other species with chronic Se toxicosis [32], but are uncommon in sheep with this condition, which in this species usually manifests only as a decrease in wool growth rates [33]. However, cardiac and hepatic atrophy, with pulmonary edema and congestion, may occur in some animals. Histologically, degeneration of cardiomyocytes may be detected in some cases, possibly associated with metabolization of Se into free radical species [30,34]. High levels of Se in wool together with a history of exposure may be of diagnostic value. There is not a specific chelating agent effective to treat selenosis available, and the main therapeutic approach consists of removing the flock from the Se sources (e.g., seleniferous area, concentrate with high levels of supplement product, etc.) [6,33,35].

### 2.6. Zinc

Sheep may be exposed to Zn salts (e.g., Zn sulfate) when they are applied in foot baths for the treatment of foot rot [6]. Cases of accidental Zn intoxication associated with the use of dietary supplements have been described, although the clinical presentation is generally acute [36]. Natural and experimental chronic Zn intoxication in sheep causes inappetance and progressive loss of condition [37]. Histologically, pancreatic fibrosis and acinar degeneration may be detected. Evidence of exposure, compatible clinical signs and lesions, and elevated Zn levels in body fluids, liver, or kidney are helpful to establish a diagnosis. Some authors consider that there is no specific treatment other than removing the flock from the presumptive Zn source [6]. CaEDTA, which is used to treat lead intoxication (see above), has been proposed as a chelating agent for Zn also [38].

### 2.7. Arsenic

Arsenic (As) is a ubiquitous element with an ample variety of species present in the environment [39,40]. Both inorganic (arsenites and arsenates) and organic As-containing compounds are considered noxious, although the toxicity tends to be higher in the former. Acute poisoning is well described and tends to be associated with marked gastroenteritis and vascular injury [6,41]. Chronic As intoxication may occur in sheep when there is persistent exposure over time associated with industrial contamination of pastures, herbicides, insecticides, fungicides, or, less frequently nowadays, some acaricidal products [6]. 

Chronically exposed animals may develop progressive ill-thrift, anorexia, anemia, diarrhea, buccal and cutaneous ulcers, dry wool, and serous atrophy of fat deposits [6,42]. In a study that compared sheep grazing in an As-contaminated area with control animals from a non-contaminated region, As-exposed sheep developed anemia and a considerable decrease in the body weight [43]. Since As progressively accumulates in a variety of tissues, increased levels may be demonstrated in liver, kidney, wool, urine, and feces [44]. The first therapeutic approach consists of identifying the As source and removing the flock from it. If the exposure has been very prolonged, the chances of recovery are limited. Substances such as 2,3-dimercaptopropanol (an As binder) or sodium thiosulfate have demonstrated effectiveness to treat As toxicosis in livestock [6,40].

### 2.8. Others

There are several intoxications with historic relevance that rarely occur nowadays. Chronic mercury poisoning occurred in sheep exposed to some pesticides, fungicides, or contaminated industrial effluents. Chronically exposed animals developed nervous signs, renal damage, anemia, and/or teeth lost. High mercury levels in the kidney, liver, and/or wool have diagnostic value. Nowadays, mercury intoxication is rare, since the use of mercury in pharmacological and agricultural products has been limited by regulatory agencies [6,45,46]. Other substances such as phenol, superphosphate fertilizers, or organophosphate pesticides tend to cause other signs that, in general, do not include severe emaciation, although they may be considered in specific situations if there is a history of exposure [6,47]. There are some drug-related processes such as overdose with the ionophore monensin that, when they occur in their milder or subacute/chronic forms, could induce loss of muscular volume and weakness due to muscle damage, thus mimicking emaciation and loss of condition [6,48].

## 3. Toxic Plants

Several components of some grasses, legumes, and shrubs contain toxins that may compromise the productivity of the animals and lead to illness and/or death, especially during periods of food scarcity. Among the toxic substances present in plants, the compounds that most frequently lead to wasting in sheep are alkaloids such as PA and swainsonine and calcinogenic glycosides [8,48,49]. This section reviews the effects of selected plants and their toxins on food intake and wasting in sheep. Table 2 summarizes relevant diagnostic aspects for some of these plant-derived compounds, and a detailed individual discussion is presented below. However, readers must be aware that poisonous plants cause a variety of clinical signs, and wasting may be just one of the consequences of the action of certain toxins on the voluntary feed intake, digestive function, and/or nutrient utilization. Toxins may also have noxious effects on cardiovascular, respiratory, musculoskeletal, and neurological systems, mainly during the chronic phases of these intoxications.

### 3.1. Pyrrolizidine Alkaloid-Containing Plants

PA-containing plants are among the most common causes of plant poisoning in livestock. More than 350 PA have been identified in more than 6000 forage species with predominance in three families: Compositae (e.g., *Senecio* spp.), Leguminosae (e.g., *Crotalaria* spp.), and Boraginaceae (e.g., *Heliotropium* spp., *Cynoglossum* spp., *Amsinckia* spp., *Echium* spp., *Trichodesma* spp., and *Symphytum* spp.) [7,9,50].

**Figure 1:**
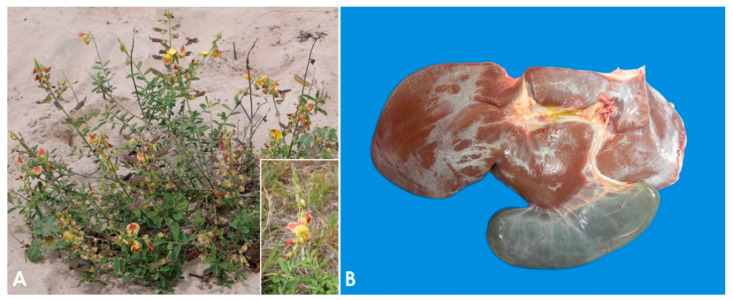
Pyrrolizidin alkaloid poisoning. (**A**) *Crotalaria retusa* plant. Inset: Detail of the flower. (**B**) Liver, sheep. Seneciosis. Moderately shrunken liver, with multifocally thickened Glisson’s capsule and distended gall bladder (Photo courtesy of Dr. Paula Giaretta).

The plants most often involved in PA toxicosis in sheep are *Heliotropium europaeum* and *Echium plantagineum* [9]. In some regions of Australia, these two plants are considered the main cause of death in sheep due to plant poisoning [50,51,52], while in Brazil the most important PA-containing plants are *Crotalaria retusa* (Figure 1A) and *Senecio brasiliensis* [53,54,55]. Other PA-containing plants associated with loss of condition in sheep in a variety of geographical locations include *Amsinckia intermedia* [56], *Crotalaria mucronata* [57], *Senecio cineraria* [58], *Senecio sanguisorbae* [59], *Senecio madagascariensis* [51], *Senecio jacobaea* [60,61], *Senecio erraticus* [62], *Heliotropium ovalifolium* [63], *Heliotropium amplexicaule* [64], *Heliotropium dasycarpum* [65], and *Echium plantagineum* [50,66,67].

PA-containing plants are non-palatable, and they only become a problem for livestock if good forage sources are unavailable and/or if they contaminate the harvested hay or other foodstuffs [8]. Furthermore, sheep are considered markedly resistant to PA poisoning because their ruminal flora is able to detoxify these compounds, and glutathione conjugation in the liver is very efficient [9,68]. For this reason, sheep grazing has been traditionally proposed as a method of biological control for several invasive species of PA-containing plants [69,70,71,72]. However, if the use of sheep with this purpose is indiscriminate, poisoning may ensue [66,67]. 

The clinical manifestations of sheep grazing PA-containing plants are variable. While some animals may present loss of appetite, pale mucous membranes, gradual loss of condition, apathy, emaciation, and death; others can live without obvious clinical signs and die suddenly [8,65]. Jaundice and varying degrees of photodermatitis characterized by crusty lesions in the ears and nose can also be observed [55,65], and stressful events such as lambing or shearing could precipitate sudden death. Clinico-pathologic changes include anemia, transient elevations in the activity of serum AST, sorbitol dehydrogenase, alkaline phosphatase, and GGT, as well as increased concentrations of serum bilirubin, bile acids, and copper [9,55,65,73].

The pathogenesis of poisoning by PA correlates with hepatic dehydration of highly reactive dehydropyrrolizidine alkaloids, which are powerful alkylating agents that react with cellular proteins and cross-link DNA, resulting in cellular dysfunction, abnormal mitosis, and tissue necrosis [9,65,74]. Ingestion of high doses of PA during a short period of time leads to acute poisoning [75]. Emaciation is generally associated with prolonged exposure, which induces two morphologically different liver patterns: 1. Phasic pattern (usually seasonal and the most common in field cases) associated with repetitive exposure to PA, which leads to hepatic atrophy with formation of regenerative nodules; multifocal, tan, 2–5 mm in diameter nodules and a distended gallbladder can be observed in necropsy. 2. Chronic pattern, which occurs with prolonged exposure to low doses of PA, and it is characterized by a firm, fibrotic and atrophic liver without nodular regeneration; the liver could look grossly normal or slightly smaller than usual, with a mild gray-yellowish discoloration due to thickening of Glisson’s capsule, and increased firmness due to parenchymal fibrosis (Figure 1B). Chronic exposure may also induce lipidosis, ketosis, or promote secondary hepatogenic Cu poisoning [8,9,65,76]. Other necropsy findings include hydropericardium, ascites, icterus, hydrothorax, and mesenteric edema.

Microscopic changes associated with PA poisoning include centrilobular hepatocyte necrosis if the intoxication is acute. In the phasic form, regenerative nodules, minimal to marked periportal and peribiliary fibrosis, bile duct hyperplasia, megalocytosis, and nuclear invaginations can be seen. In the chronic or prolonged exposure-related form, variable hepatocyte atrophy, slight bile duct hyperplasia, and minimal periportal fibrosis are observed [8,9,55,65,74,77].

### 3.2. Indolizidine Alkaloids (Swainsonine-Containing Plants)

**Figure 2:**
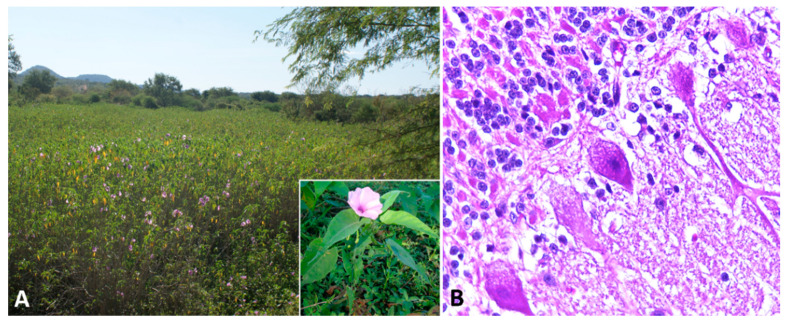
Swainsonine-containing plant poisoning. (**A**) *Ipomoea carnea* subsp. *fistulosa*-invaded area. Inset: Detail of the plant and flower. (**B**) Cerebellum, sheep. Slight cytoplasmic vacuolization of Purkinje cells.

Indolizidine alkaloids are found in several species of three genera in the Fabaceae (Fabales) family, including *Astragalus* spp., *Oxytropis* spp., and *Swainsona* spp. Swainsonine, is the main bioactive principle found in these genera of plants. In addition, swainsonine has been documented in two other plant families, the Convolvulaceae (Solanales) and the Malvaceae (Malvales) [8]. Toxic potential is attributed to several species, including poison pea (*Swainsona* spp.), locoweeds (*Astragalus* spp. and *Oxytropis* spp.), broomweed (*Sida carpinifolia*) and several species belonging to the morning glory family (*Ipomoea* spp.; Figure 2A) [78,79,80,81]. The alkaloid is found in every part of the plant, with highest concentrations in the leaves, flowers, and seeds [79]. However, many of the species within these families are nontoxic and considered important forage resources. 

Swainsonine is a potent inhibitor of two lysosomal enzymes, alpha-D-mannosidase and Golgi mannosidase II, which are important in the metabolism of saccharides and the formation of glycoproteins. The inhibition of alpha-mannosidase causes cells to accumulate oligosaccharides, whereas the inhibition of Golgi mannosidase II affects the normal structure of glycoproteins [82]. As a result, oligosaccharide glycosylated proteins accumulate in the cells of the brain and many other organs, interfering with normal cellular function [83,84]. Other similar polyhydroxylated indolizidines with different stereochemical configurations, such as castanospermine and calystegines, have been identified in several plants and are specific inhibitors of other enzymes [8,49,85].

Sheep intoxicated with indolizidine alkaloids-containing plants are lethargic, anorexic, reluctant to move, and may show neurologic signs that range from subtle anxiety to seizures [80,86,87]. Loss of condition and emaciation are typical and may also occur in situations of plentiful feed [86,88,89]. In an experimental study, sheep that received *Oxytropis sericea* refused up to 50% of their ration after 3 weeks, which resulted in significantly reduced weight gains [90].

Neurogenic anorexia, inability to eat properly, and impairment of glycosylation and secretion of intestinal and pancreatic exocrine enzymes, with subsequent poor digestion and absorption, are the main mechanisms related to weight loss or reduced weight gains in poisoned sheep [85,87,90]. Sheep become cachectic and they develop more severe neurologic signs after continuous exposure to indolizidine alkaloids for weeks or months [8]. Other clinical signs such as infertility, reproductive failure, and abortions are also reported. Ewes poisoned later in gestation often give birth to small and weak lambs [79].

Affected sheep normally have no prominent gross lesions other than emaciation with marked loss of adipose tissue. Histologically, widespread cytoplasmic vacuolation in different locations, especially epithelia and nervous tissues, may be detected. Neuronal vacuolation is most severe in cerebellar Purkinje cells (Figure 2B) and large neurons of the basal ganglia [86,87,88,90,91,92]. Purkinje cells also present axonal dystrophy and abnormal neuritic processes, which may include meganeurite formation at the axonal hillock, aberrant synapses, and dendritic outgrowths. Some cerebellar Purkinje cells are lost (empty axonal baskets) and undergo subsequent axonal degeneration and spheroid formation [8,93].

Swainsonine-containing plant poisoning may be tentatively diagnosed by demonstrating the presence of swainsonine in the serum, coupled with decreased serum alpha-mannosidase activity [94]. Lectin histochemistry in liver biopsies could be an effective diagnostic method, even in animals without neurological signs [95]. An accurate postmortem diagnosis may be done by demonstrating the presence of characteristic cytoplasmic vacuoles in the cerebellar Purkinje cells, lymphocytes, liver, thyroid gland, and several other tissues.

### 3.3. Calcinogenic Glycoside-Containing Plants

**Figure 3:**
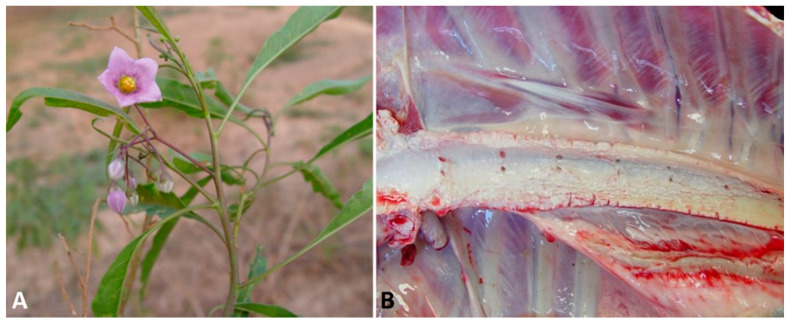
Calcinogenic glycoside-containing plant poisoning. (**A**) *Solanum malacoxylon* plant. (**B**) Aorta, sheep. Mineralization due to *Solanum malacoxylon* intoxication (Photos courtesy of Dr. Edson Moleta Colodel).

Sheep grazing calcinogenic plants develop a progressive debilitating disease with widespread mineralization of soft tissues called enzootic calcinosis. The main species of calcinogenic plants that affect sheep are *Cestrum diurnum* in southern United States [7], *Trisetum flavescens* in Bavarian and Austrian Alps [96,97], *Solanum malacoxylon* (Figure 3A) in Argentina and Uruguay [98], *Solanum escuriali* in Australia [99], *Solanum verbascifolium* in South Africa [100], *Nierembergia veitchii* in Brazil [101,102], and *Nierembergia repens* and *Nierembergia rivularis* in Uruguay [103]. In some countries such as Israel, India and Central Western Brazil, outbreaks caused by undetermined calcinogenic plants occurred in sheep [98,104]. 

The leaves of these calcinogenic plants contain a glycoside related to 1,25-dihydroxycholecalciferol (calcitriol) or a calcitriol-like compound with noxious biologic activity. Calcitriol is the active form of vitamin D (cholecalciferol) and acts by increasing calcium absorption from the gastrointestinal tract and bone resorption, and by decreasing calcium excretion in the kidneys [79]. Intoxication by these plants produces rapid wasting and marked elevations of the calcium and phosphate levels in the blood [79], causing metastatic calcification in the heart, aorta, lungs, kidneys, and joints. As the renal metastatic calcification progresses, there is an increase in blood urea nitrogen and creatinine on serum chemistry analyses. Radiology may aid in revealing calcification in several organs, including the vascular walls of the limbs [79,96]. 

Poisoning is progressive, and the first clinical signs observed are anorexia, depression, weakness, weight loss, infertility, cardiac arrhythmias, cachexia, stiffness, impaired stilted gait, kyphosis, and recumbence. Musculoskeletal abnormalities, including slight flexion of the forelimbs while walking, abnormally straight hind limbs, and/or knee walking, may be severe in some sheep. Increased respiratory rate, abdominal distension due to ascites, and reduced rumen motility are also described. Death generally occurs after a protracted clinical course [96,102,103,104]. The majority of these signs are related to hypercalcemia and subsequent soft tissue calcification. Death may occur as a result of emaciation and weakness, as well as secondary to cardiac and/or pulmonary insufficiency [79,105].

At necropsy, depletion of fat deposits is noted, and mineralization can be visualized as aggregates of a gritty, white to tan material on the surfaces of several organs and tissues, particularly in the aorta (Figure 3B), valvular and mural endocardium, lungs, and kidneys [97]. Mineralization may be also observed in the uterine, omasal, ruminal, and reticular serosae [102]. Histologically, systemic tissue mineralization is readily detected as fine, granular, basophilic deposits, and the extent of tissue damage is related to the severity and duration of the hypercalcemia. In the arteries, mineralization is frequently observed in the subintimal media, in which chondroid and osseous metaplasia occasionally occur. Mineral may be found in the renal parenchyma, pulmonary alveolar septa, bronchiolar epithelium, endocardium, gastric and intestinal musculature, and other tissues. In response to chronic hypercalcemia, the thyroid C cells often become hyperplastic and the parathyroid gland may undergo atrophy [8,79,96,97,102,103].

A tentative diagnosis of enzootic calcinosis can be made by measuring serum levels of 1,25-dihydroxycholecalciferol, which may be detectable in recently exposed animals [79]. Demonstration of mineral deposits in tissues by ultrasonography is also a valid in vivo approach [96]. Postmortem visualization of mineral deposits in the heart, aorta, lung, or kidney, either at necropsy or microscopically, coupled with a history of calcinogenic plant exposure, is probably the most reliable diagnostic method. The prognosis is unfavorable once extensive calcification has occurred, since there is no treatment. Prevention and control may be attempted by removing these weeds from the grazing areas.

### 3.4. Nitrate-Containing Plants

Nitrates are present in many plants, and the most common source of poisoning in sheep is found in crops or several weeds that tend to accumulate these compounds. Forage crops from soils that were heavily fertilized may also accumulate high levels of nitrate. Additionally, hay or fresh cut forage fed to animals as green-chop may be also a source of nitrate poisoning [18,106,107]. Fodder that contains from 1–1.5% potassium nitrate on a dry matter basis may cause acute poisoning in ruminants [108].

The list of plants containing nitrates is extensive and includes: Pigweed (*Amaranthus* spp.); tarweed (*Amsinckia* spp.); ragweed (*Ambrosia* spp.); oats (*Avena sativa*); beets (*Beta vulgaris*); rutabaga, rape, broccoli, and turnip (*Brassica* spp.); lamb’s quarters (*Chenopodium* spp.); Canada thistle (*Cirsium arvense*); bindweed (*Convolvulus* spp.); jimsonweed (*Datura* spp.); wild carrot or Queen Anne’s lace (*Daucus carota*); goosegrass (*Eleusine indica*); thoroughwort (*Eupatorium purpureum*); soybean (*Glycine max*); purple cudweed (*Gnaphalium purpureum*); sunflower (*Helianthus annuus*); barley (*Hordeum vulgare*); sweet potato (*Ipomoea batatas*); firebush (*Kochia* spp.); prickly lettuce (*Lactuca serriola*); flax (*Linum* spp.); small mallow (*Malva parviflora*); sweet clover (*Melilotus officinalis*); panic grass (*Panicum capillare*); smartweed (*Polygonum* spp.); dock (*Rumex* spp.); Russian thistle (*Salsola iberica*); rye (*Secale cereale*); nightshades and potatoes (*Solanum* spp.); goldenrods (*Solidago* spp.); Johnson grass, Milo, and Sudan grass (*Sorghum* spp.); Chickweed (*Stellaria media*); puncture vine (*Tribulus terrestris*); wheat (*Triticum sativum*); stinging nettle (*Urtica dioica*); golden crownbeard (*Verbesina encelioides*); and Corn (*Zea mays*) [18]. The concentration of nitrates in these plants varies considerably.

The pathogenesis, clinical presentation and lesions associated with intoxications by nitrate-containing plants are similar to those described above for other nitrate sources (see Section 2.2.2 Nitrates and Nitrites) and are mostly related to methemoglobinemia. Diagnostic approaches are also equivalent, and once the nitrate-containing plant source has been identified, the flock should be rapidly removed from it. Treatment with methylene blue may also be attempted [6,79,109,110,111].

## 4. Conclusions

This review highlights the broad variety of toxic organic/inorganic compounds and plants that may be involved in states of emaciation, wasting, and loss of productivity in sheep. These conditions should be thus considered as differential diagnoses, especially in those animals and flocks where the classic infectious and parasitic wasting diseases have been ruled out [1,2,3,4]. Diagnosis may be fastidious in many cases, since lesions could be minimal and/or unspecific, and a good anamnesis focused on revealing a history of exposure should be the cornerstone of the diagnostic work-up. Once the diagnosis is established, removing the animals from the toxic source is the first step to take.

## Figures and Tables

**Table 1:** Treatments protocols for different toxicants.

Toxicant	Treatment	Dose and Mode of Administration
Copper	Ammonium tetrathiomolybdate	1.68 mg/kg BW IV (3 times in 5 d) 3.4 mg/kg BW SC (3 alternate d)
Nitrates/nitrites	Methylene blue 4%	10 mg/kg BW, IV infusion
Fluoride/Fluorine	Monoacetin (acute cases)	0.55 mL/kg BW IM every 1/2 h, several hours
	Ca and Al compounds added to the ration	
Lead	CaEDTA	75 mg/kg IV, slow infusion, daily during the first 48 h
Selenium	No specific chelation therapy available	-
Zinc	CaEDTA	100 mg/kg IV or SQ, into four doses/day for 3 d
Arsenic	2,3-dimercaptopropanol	23 mg/kg every 4 h for 2 d, repeat every 6 h for 2 d, repeat every 12 h for 2 d
	Sodium thiosulfate	30–40 mg/kg 20% solution IV, repeated every 12 h until clinical improvement

d: day; h: hour; CaEDTA: Calcium disodium ethylenediaminetetraacetic acid; BW: body weight; IM: intramuscular; IV: intravenous; SQ: subcutaneous; Ca: calcium; Al: aluminum.

**Table 2:** Diagnostically relevant lesions and ancillary tests for plant-derived toxicants.

Toxicant	Gross Lesions *	Histological Lesions	Ancillary Tests
Pyrrolizidin alkaloid	Atrophic and firm liver with/without nodular regeneration	Fibrosis Bile duct hyperplasia Megalocytosis Nuclear invaginations Hepatocyte atrophy	-
Indolizidine alkaloid (swainsonine)	-	Cytoplasmic vacuolization in epithelial and neural cells	Lectin histochemistry in liver Increased serum swansonine Decreased serum alpha-mannosidase activity
Calcinogenic glycoside	Gritty mineral deposits in tissues	Basophilic mineral deposits	Elevated serum 1,25-dihydroxycholecalciferol
Nitrates/nitrites	Brownish discoloration of carcass and organs Brown and dense blood	-	Nitrates/nitrites concentration in ocular fluid

* All toxicants may induce different degrees of emaciation.

## Data Availability

Data sharing not applicable.
